# Supplementary material for: PITX2 dosage-dependent changes in pacemaker cell state underlie sinus node dysfunction and atrial arrhythmias
Source: Nat Commun. 2025 Dec 5;16:11197. doi: 10.1038/s41467-025-66959-3 (PMC12712101; doi:10.1038/s41467-025-66959-3)
Supplement: Supplementary file 1 — Supplementary Information [file 41467_2025_66959_MOESM1_ESM.pdf]

Supplementary Information

**Baseline ECG phenotypes**	***WT***	***delB/+***	***delB/delB***
**n**	33	27	45
Negative first P wave deflection (Lead I)	1 (3%)	11 (41%) $	35 (78%) *,#
One P wave shape – Regular rhythm	29 (88%)	21 (78%)	11 (24%) *,#
One P wave shape – Sinus pauses	0 (0%)	0 (0%)	6 (13%) *,#
Two P waves shape – Regular rhythm	0 (0%)	2 (6%)	7 (16%) *
Two P waves shape – Sinus pauses	0 (0%)	1 (4%)	13 (29%) *,#
Different P wave shape after breathing	0 (0%)	0 (0%)	2 (4%)
Junctional rhythm	0 (0%)	1 (4%)	6 (13%) *
Short coupled atrial ectopy (followed by AV-block or AV conduction)	3 (9%)	1 (4%)	1 (2%)
Premature ventricular contractions	1 (3%)	1 (4%)	1 (2%)

Supplementary table 1. Electrocardiographic phenotypes in *wild-type* (*WT*), *delB/+* and *delB/delB* adult mice. Chi-square tests: $*p*<0.05 *delB/+* vs *WT*, **p*<0.05 *delB/delB* vs *WT*, #*p*<0.05 *delB/delB* vs *delB/+*.

**Baseline ECG (LeadI)**	**Heart rate (BMP)**	**PR interval (ms)**	**P duration (ms)**	**QRS duration (ms)**	**QT interval (ms)**
WT (n=33)	462 ± 55	33 ± 3	19.5 ± 0.2	8.7 ± 0.5	48 ± 6
delB/+ (n=27)	433 ± 77	33 ± 3	19.8 ± 0.1	8.8 ± 0.7	51 ± 9
delB/delB (n=45)	366 ± 73 *,#	33 ± 3	20 ± 0.2	8.9 ± 1.3	53 ± 7
One-way ANOVA	p<0.0001	NS	NS	NS (non-parametric)	NS

Supplementary table 2. Baseline electrocardiographic parameters of *wild-type* (*WT*), *delB/+* and *delB/delB* adult mice. ECG parameters were assessed in consecutive complexes of the dominant rhythm (excluding atrial ectopy). Statistics: One-way ANOVA for each parameter (non-parametric if mentioned), with Bonferonni's post-hoc tests: *p<0.0001 *delB/delB* vs *WT*, #p=0.0004 *delB/delB* vs *delB/+*.

**ECG phenotypes before and after ANS block**	***WT***	***WT* ANS block**	***delB/+***	***delB/+* ANS block**	***delB/delB***	***delB/delB* ANS block**
**n**	17	17	18	18	24	24
Negative first P wave deflection (Lead I)	1 (6%)	1 (6%)	7 (39%)	2 (11%)	21 (88%)	15 (63%)
One P wave shape – Regular rhythm	14 (83%)	16 (94%)	16 (89%)	18 (100%)	7 (29%)	17 (71%) *
One P wave shape – Sinus pauses	0 (0%)	0 (0%)	0 (0%)	0 (0%)	1 (4%)	1 (4%)
Two P waves shape – Regular rhythm	0 (0%)	0 (0%)	1 (6%)	0 (0%)	6 (25%)	2 (8%)
Two P waves shape – Sinus pauses	0 (0%)	0 (0%)	1 (4%)	0 (0%)	4 (17%)	1 (4%)
Different P wave shape after breathing	0 (0%)	0 (0%)	0 (0%)	0 (0%)	1 (4%)	0 (0%)
Junctional rhythm	0 (0%)	0 (0%)	1 (4%)	0 (0%)	3 (13%)	3 (13%)
Short coupled atrial ectopy (followed by AV-block or AV conduction)	2 (11%)	1 (6%)	1 (6%)	0 (0%)	1 (4%)	0 (0%)
Premature ventricular contractions	1 (6%)	0 (0%)	1 (4%)	0 (0%)	1 (4%)	0 (0%)

Supplementary table 3. Electrocardiographic phenotypes in *wild-type* (*WT*), *delB/+* and *delB/delB* adult mice before and after Autonomic Nervous System blockade (ANSblock). Chi-square tests: *p<0.05 ANSblock vs no ANSblock in *delB/delB*.

ECG (LeadI)	Heart rate (BMP)	PR interval (ms)	P duration (ms)	QRS duration (ms)	QT interval (ms)
WT (n=17)	465 ± 46	32.3 ± 3	19.4 ± 2	8.7 ± 8	52.1 ± 6
WT ANSblock (n=17)	358 ± 45 *	34.6 ± 3 *	19.9 ± 3	8.6 ± 1	59 ± 8 *
delB/+ (n=18)	433 ± 81	33.6 ± 3	19.7 ± 2	8.9 ± 0.8	51,5 ± 8
delB/+ ANSblock (n=18)	313 ± 50 *	35.7 ± 4 *	17.6 ± 4 *,#	11.3 ± 2.2 *,#	68.6 ± 13 *,#
delB/delB (n=24)	381 ± 78 #,$	33 ± 4	19.7 ± 2	8.3 ± 1	51 ± 6
delB/delB ANSblock (n=24)	224 ± 65 *,#	33.6 ± 5	17.8 ± 3 *,$	10.2 ± 2 *,#,$	61.2 ± 8 *,$
Two-way ANOVA	Gen 0.0001<br>ANS <0.0001<br>GenxANS NS	Gen NS<br>ANS <0.0001<br>GenxANS NS	Gen NS<br>ANS 0.0033<br>GenxANS 0.0122	Gen 0.0016<br>ANS <0.0001<br>GenxANS 0.0002	Gen NS<br>ANS <0.0001<br>GenxANS 0.0065

Supplementary table 4. Electrocardiographic phenotypes in *wild-type* (*WT*), *delB/+* and *delB/delB* adult mice before and after Autonomic Nervous System blockade (ANSblock). ECG parameters were assessed in consecutive complexes of the dominant rhythm (excluding atrial ectopy). Statistics: Two-way ANOVA with repeated measures (factors: Genotype (Gen), ANSblock (ANS)) for each parameter (non-parametric mixed-effects analysis if mentioned), *$p<0.05$ no ANSblock vs ANSblock in the same genotype, # $p<0.05$ different from *WT* of the same conduction, $ different from *delB/+* of the same condition.

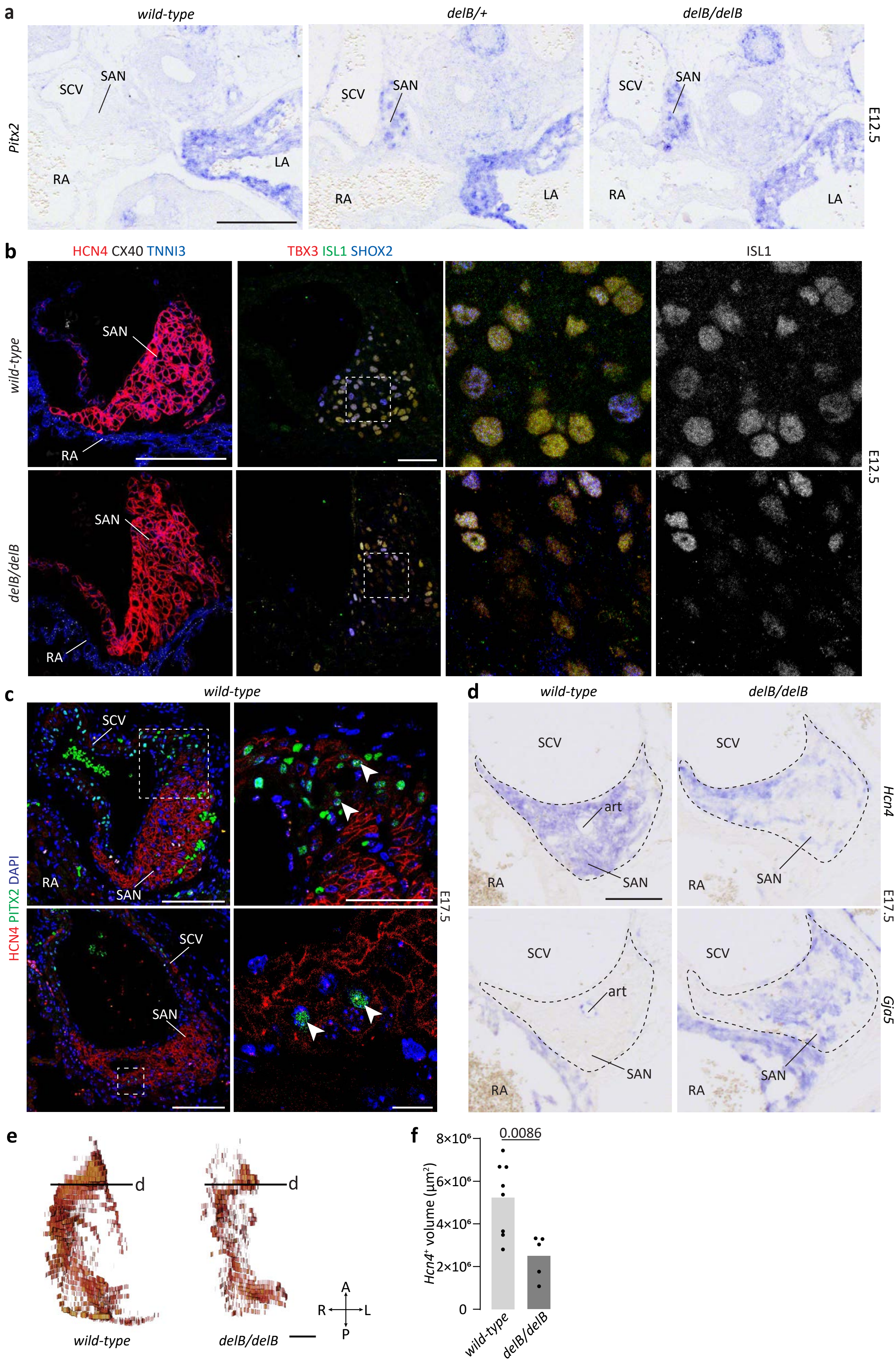
a
wild-type
delB/+
delB/delB
Pitx2
SCV
SAN
RA
LA
E12.5
b
HCN4 CX40 TNNI3
TBX3 ISL1 SHOX2
ISL1
wild-type
delB/delB
E12.5
c
wild-type
HCN4 PITX2 DAPI
E17.5
d
wild-type
delB/delB
art
Hcn4
Gja5
E17.5
e
wild-type
delB/delB
A
P
R
L
f
0.0086
8×10⁶
6×10⁶
4×10⁶
2×10⁶
0
Hcn4+ volume (µm³)

Supplementary Figure 1. Ectopic *Pitx2* expression and loss of PC-associated gene expression in the developing *delB* SAN. a. *In situ* hybridization shows that *Pitx2* clearly demarcates the LA from the RA in *wild-type* (n=2), *delB/+* (n=3), and *delB/delB* (n=2) hearts and that *Pitx2* is ectopically expressed in the SAN in *delB/+* and *delB/delB* mice at E12.5. Scale is 200 µm. **b**. Immunostaining shows the expression of downstream functional SAN marker HCN4 in the *wild-type* SAN (n=5) and shows that its expression in the *delB/delB* SAN (n=4) at E12.5 is unaffected. In adjacent sections, the expression of PC transcription factors ISL1, TBX3, and SHOX2 overlaps with the *wild-type* HCN4+ domain (n=2). However, a graded reduction in ISL1, TBX3 and SHOX2 expression can be detected in the *delB/delB* SAN (n=2) at this developmental stage. Scale is 100 µm and 10 µm. **c.** A PITX2+ region is present in the dorsal SAN head domain adjacent to the SCV myocardium in the *wild-type* E17.5 heart (n=3), scale is 100 µm and 50 µm. Sporadic PITX2+ nuclei are also occasionally found in the core of the SAN head domain at this developmental stage, scale is 100 µm and 10 µm. **d**. *In situ* hybridization shows loss of *Hcn4* expression and concomitant activation of *Gja5* expression in adjacent sections in the E17.5 SAN (*wild-type*, n=8; *delB/delB*, n=5). Scale is 100 µm. **e**. Representative 3D reconstructions of the *Hcn4*+ domain in the E17.5 RA depicted in panel d. Scale is 200 µm. **f.** Quantification of *Hcn4* expression domain at E17.5 in *wild-type* (n=8) and *delB/delB* (n=5) reveals a 2-fold reduction in the *Hcn4* expression volume in *delB/delB* RA (p= 0.0086; unpaired two-tailed T-test). Source data are provided in the Source Data file. SAN, sinus node; SCV, superior caval vein; LA, left atrium; RA, right atrium; E, embryonic day; PC, pacemaker cardiomyocyte; A, anterior; L, left; P, posterior; R, right.

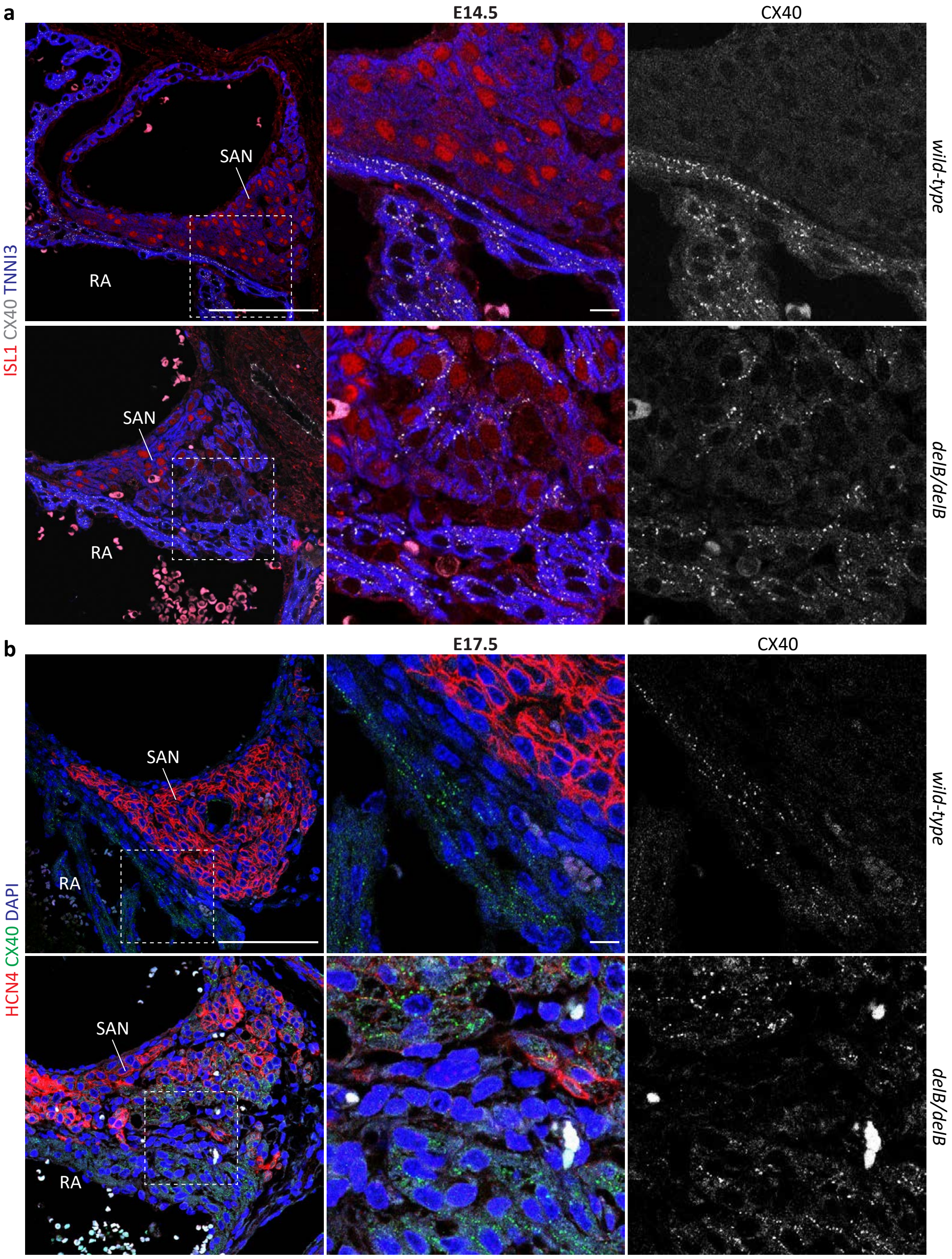

a
E14.5
CX40
SAN
RA
ISL1 CX40 TNNI3
wild-type
SAN
RA
delB/delB
b
E17.5
CX40
SAN
RA
HCN4 CX40 DAPI
wild-type
SAN
RA
delB/delB

Supplementary Figure 2. Ectopic CX40 expression in the *delB/delB* SAN. a. Immunostaining demonstrating how CX40 clearly demarcates the boundary between the *wild-type* (n=5) RA and SAN at E14.5. In the E14.5 *delB/delB* (n=4) SAN, clusters of PCs have lost ISL1 expression and ectopically express CX40. Scale is 100 and 10 µm. **b.** Immunostainings show that CX40 continues to demarcate the boundary between the *wild-type* (n=3) RA and SAN at E17.5 and that CX40 is ectopically expressed in the *delB/delB* (n-4) $HCN4^{low}$ SAN subdomain. Scale is 100 and 10 µm. SAN, sinus node; RA, right atrium; E, embryonic day.

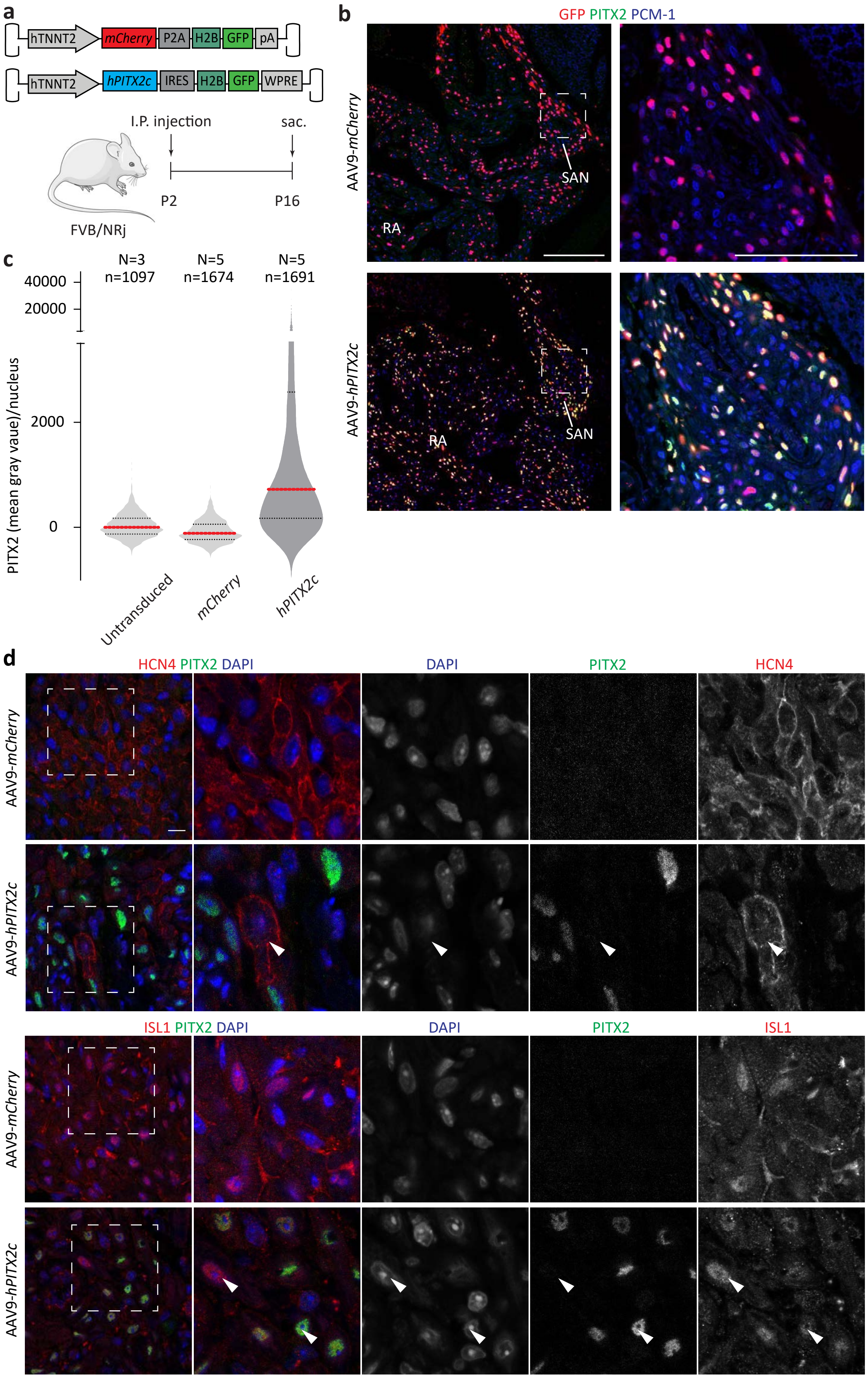

a
hTNNT2
mCherry
P2A
H2B
GFP
pA
hTNNT2
hPITX2c
IRES
H2B
GFP
WPRE
I.P. injection
sac.
P2
P16
FVB/NRj
b
GFP PITX2 PCM-1
AAV9-mCherry
SAN
RA
AAV9-hPITX2c
SAN
RA
c
N=3 n=1097
N=5 n=1674
N=5 n=1691
40000
20000
2000
0
PITX2 (mean gray vaue)/nucleus
Untransduced
mCherry
hPITX2c
d
HCN4 PITX2 DAPI
DAPI
PITX2
HCN4
AAV9-mCherry
AAV9-hPITX2c
ISL1 PITX2 DAPI
DAPI
PITX2
ISL1
AAV9-mCherry
AAV9-hPITX2c

Supplementary Figure 3. PITX2 overexpression in postnatal, differentiated PCs causes broad loss of PC identity in the SAN. a. *Wild-type* mice were intraperitoneally injected at P2 with AAV9-Myo4A expression viruses driving *mCherry* (n=5) or *PITX2* (n=5), and *EGFP* expression. Image provided by Servier Medical Art (https://smart.servier.com), licensed under CC BY4.0. (https://creativecommons.org/licenses/by/4.0/). **b.** 14 days post-transduction, the pups were sacrificed and a high cardiomyocyte-specific transduction efficiency was determined by immunostaining for GFP, PITX2 and PCM-1 in mice that were transduced with the AAV9-Myo4a-*mCherry* control and AAV9-Myo4A-*PITX2*. **c.** Quantification of mean gray value per nucleus in the untransduced (N=3; 1097 nuclei), AAV9- *mcherry* (N=5, 1674 nuclei) and AAV9-*PITX2c* (N=5, 1691 nuclei) P14 SAN demonstrates the degree of PITX2 expression in the AAV9-*PITX2c* SAN (p<0.0001; two-sided Kolmogorov-Smirnov test). **d.** Immunostaining for HCN4, PITX2 and DAPI and ISL1, PITX2 and DAPI show that PITX2 overexpression in the postnatal SAN is sufficient to deplete PC identity. Scale is 10 µm. Source data are provided in the Source Data file. I.P., intraperitoneal; sac., sacrifice; P, postnatal day; SAN, sinus node; RA, right atrium.

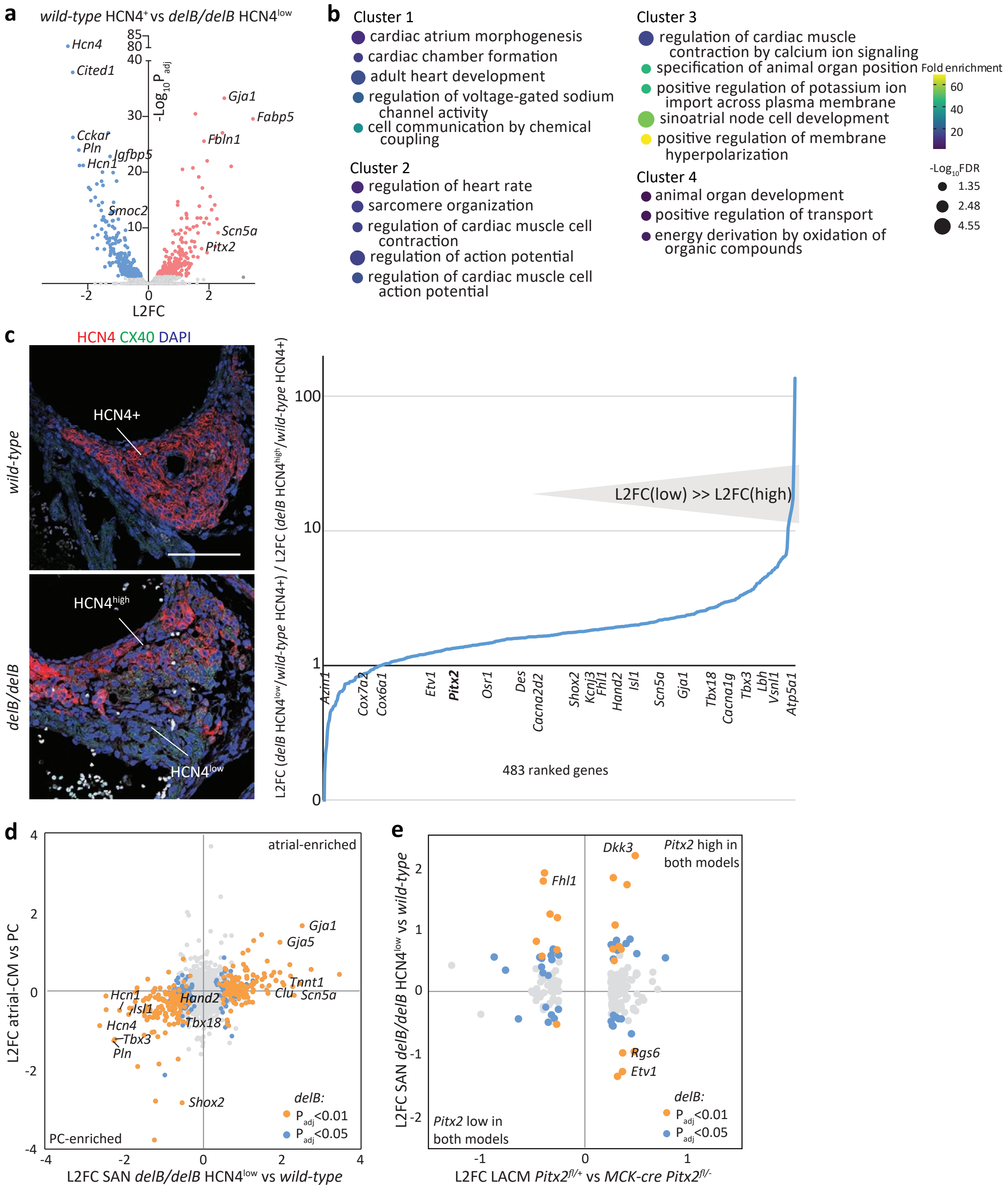
a
wild-type HCN4+ vs delB/delB HCN4low
Hcn4
Cited1
Gja1
Fabp5
Fbln1
Cckar
Pln
Igfbp5
Hcn1
Smoc2
Scn5a
Pitx2
-Log10 Padj
L2FC
b
Cluster 1
cardiac atrium morphogenesis
cardiac chamber formation
adult heart development
regulation of voltage-gated sodium channel activity
cell communication by chemical coupling
Cluster 2
regulation of heart rate
sarcomere organization
regulation of cardiac muscle cell contraction
regulation of action potential
regulation of cardiac muscle cell action potential
Cluster 3
regulation of cardiac muscle contraction by calcium ion signaling
specification of animal organ position
positive regulation of potassium ion import across plasma membrane
sinoatrial node cell development
positive regulation of membrane hyperpolarization
Cluster 4
animal organ development
positive regulation of transport
energy derivation by oxidation of organic compounds
Fold enrichment
-Log10FDR
c
HCN4 CX40 DAPI
wild-type
delB/delB
HCN4+
HCN4high
HCN4low
L2FC (delB HCN4low/wild-type HCN4+) / L2FC (delB HCN4high/wild-type HCN4+)
L2FC(low) >> L2FC(high)
483 ranked genes
Azin1 Cox7a2 Cox6a1 Etv1 Pitx2 Osr1 Des Cacna2d2 Shox2 Kcnj3 Fhl1 Hand2 Isl1 Scn5a Gja1 Tbx18 Cacna1g Tbx3 Lbh Vsnl1 Atp5a1
d
atrial-enriched
PC-enriched
L2FC atrial-CM vs PC
L2FC SAN delB/delB HCN4low vs wild-type
Gja1 Gja5 Tnnt1 Clu Scn5a Hcn1 Isl1 Hand2 Hcn4 Tbx18 Tbx3 Pln Shox2
delB:
Padj<0.01
Padj<0.05
e
Pitx2 high in both models
Pitx2 low in both models
Dkk3 Fhl1 Rgs6 Etv1
L2FC SAN delB/delB HCN4low vs wild-type
L2FC LACM Pitx2fl/+ vs MCK-cre Pitx2fl/-

Supplementary Figure 4. Transcriptional consequences of *Pitx2* expression in the SAN. a. Expression analysis of the *wild-type* HCN4+ SAN and *delB/delB* HCN4^{low} subdomains shows differential gene expression when comparing *wild-type* and *delB/delB* HCN4^{low} PCs. Blue dots highlight genes that are significantly downregulated in the *delB/delB* HCN4^{low} population and red dots highlight genes upregulated in the *delB/delB* HCN4^{low} population (p_{adj}<0.05). Wald test corrected for multiple comparisons using the Benjamini-Hochberg method. **b.** Gene ontology analysis on each identified cluster shown in Figure 3f illustrates that differentially expressed genes in each cluster are enriched for terms associated with altered ion handling, metabolism and loss of PC-associated phenotype. **c**. Immunostainings showing the HCN4+ *wild-type* SAN and the HCN4^{low} and HCN4^{high} subdomains in the *delB/delB* E17.5 SAN, scale is 100 µm. Ranking relative gene expression upon comparing differentially expressed genes in *delB/delB* HCN4^{low} vs *wild-type* HCN4+ SAN and those *delB/delB* HCN4^{high} vs *wild-type* HCN4+ SAN indicates that most genes react stronger to higher levels of PITX2 than lower levels. **d**. Scatterplot comparing the L2FC of differentially expressed genes when comparing the *delB/delB* HCN4^{low} domain with the *wild-type* SAN (orange, p_{adj}<0.01 and blue, p_{adj}<0.05) and those when comparing *Pitx2$^{fl/+}$* with *Pitx2* haploinsufficient (*MCK-cre Pitx2$^{fl/-}$*) LA CMs[34] shows minor overlap in response to altered PITX2 dosage in the SAN and LA. **e.** Scatterplot comparing the differences in gene expression in *wild-type* E16.5 PCs and atrial CMs[72] with those in the E17.5 *delB/delB* HCN4^{low} SAN and the *wild-type* HCN4+ SAN (orange, p_{adj}<0.01 and blue, p_{adj}<0.05) demonstrates the transcriptomic overlap between atrial CMs and PITX2+ *delB/delB* HCN4^{low} PCs. Wald test corrected for multiple comparisons using the Benjamini-Hochberg method. Exact *p* and p_{adj} values and L2FC values are listed in Supplementary Data 1-3. Source data are provided in the Source Data file. SAN, sinus node; PCs, pacemaker cardiomyocytes; Log_2Fold-change, L2FC; LA, left atrium; CM, cardiomyocytes; FDR, false discovery rate; L2FC, Log_2Fold-change.

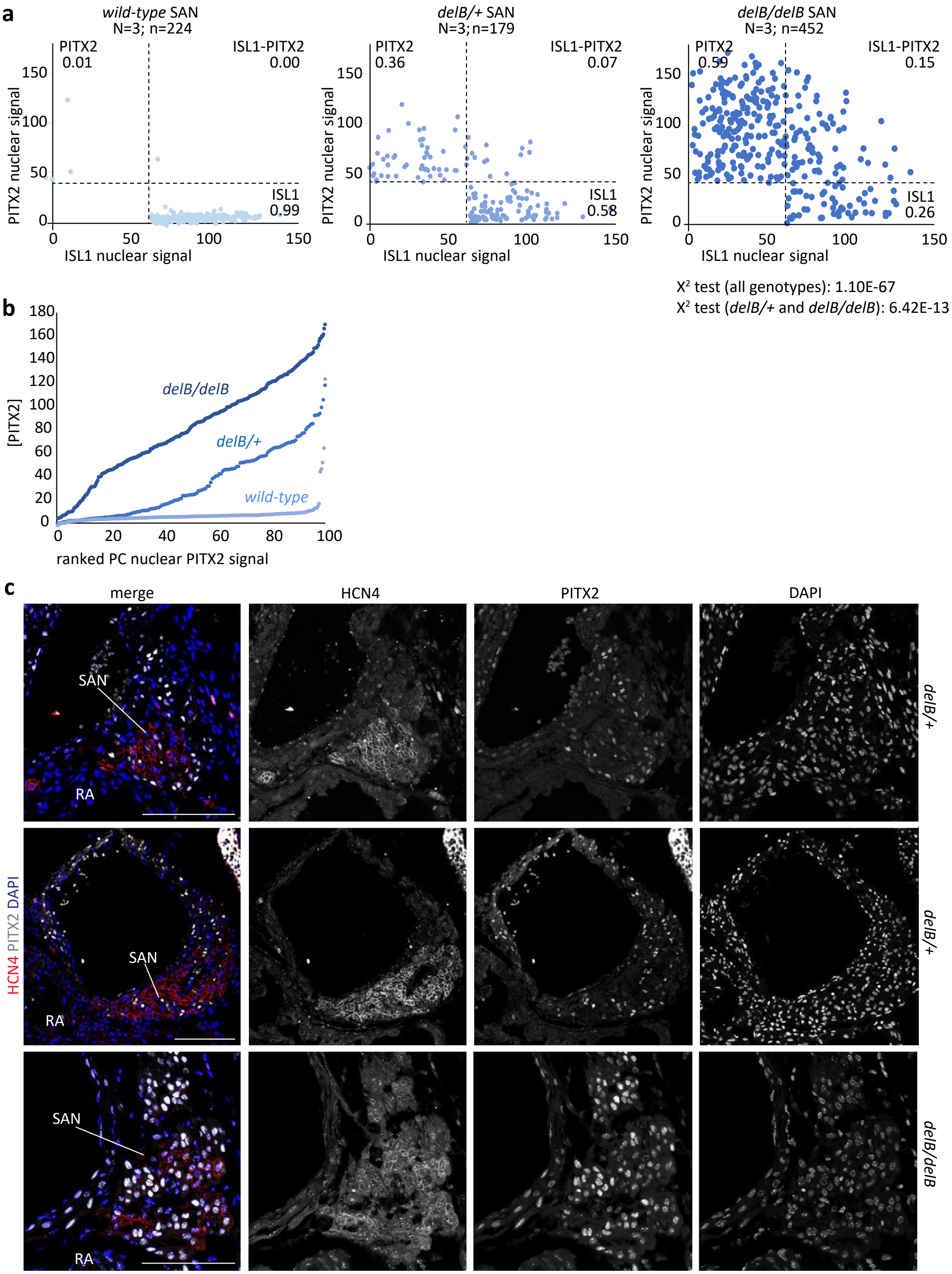
a
wild-type SAN
N=3; n=224
PITX2
0.01
ISL1-PITX2
0.00
ISL1
0.99
PITX2 nuclear signal
ISL1 nuclear signal
delB/+ SAN
N=3; n=179
PITX2
0.36
ISL1-PITX2
0.07
ISL1
0.58
delB/delB SAN
N=3; n=452
PITX2
0.59
ISL1-PITX2
0.15
ISL1
0.26
X² test (all genotypes): 1.10E-67
X² test (delB/+ and delB/delB): 6.42E-13
b
[PITX2]
delB/delB
delB/+
wild-type
ranked PC nuclear PITX2 signal
c
merge
HCN4
PITX2
DAPI
SAN
RA
HCN4 PITX2 DAPI
delB/+
delB/+
delB/delB

Supplementary Figure 5. PITX2 and ISL1 expression in the prenatal *delB* SAN. a. Scatterplots showing PITX2 and ISL1 normalized mean gray value per nucleus in the *wild-type, delB/+* and *delB/delB* E17.5 SAN. **b.** Ranked PC nuclear mean gray value for PITX2 in the *wild-type, delB/+* and *delB/delB* E17.5 SAN, showing a greater proportion of PITX2+ nuclei in the *delB/delB* SAN vs the *delB/+* SAN. **c.** HCN4 and PITX2 expression in the *delB/+* and *delB/delB* SAN reveals clusters of $HCN4^{low}$/PITX2+ and $HCN4^{high}$/PITX2- nuclei in the SAN. Scale is 100 µm. Source data are provided in the Source Data file. SAN, sinus node; PC, pacemaker cardiomyocyte.

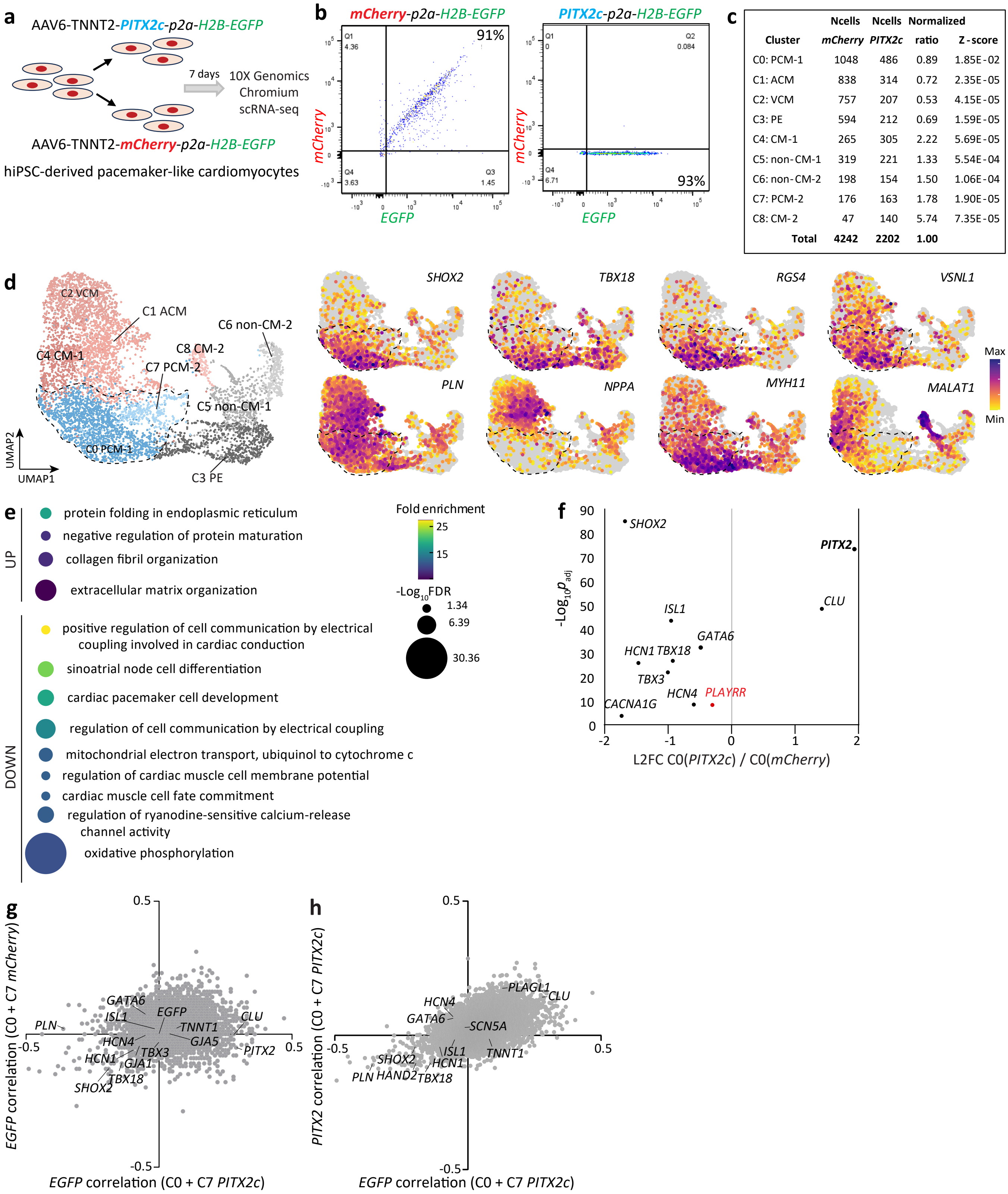

Cluster	Ncells mCherry	Ncells PITX2c	Normalized ratio	Z-score
C0: PCM-1	1048	486	0.89	1.85E-02
C1: ACM	838	314	0.72	2.35E-05
C2: VCM	757	207	0.53	4.15E-05
C3: PE	594	212	0.69	1.59E-05
C4: CM-1	265	305	2.22	5.69E-05
C5: non-CM-1	319	221	1.33	5.54E-04
C6: non-CM-2	198	154	1.50	1.06E-04
C7: PCM-2	176	163	1.78	1.90E-05
C8: CM-2	47	140	5.74	7.35E-05
Total	4242	2202	1.00	

Supplementary Figure 6. scRNA seq of hiPSC-PCs that overexpress *PITX2c*. a. Schematic overview of the workflow. b**.** hiPSC-PCs were transduced with AAV6 viruses to achieve *mCherry* and *PITX2c* overexpression. Flow cytometry plots show a >90% transduction efficiency. **c**. The contribution of cells transduced with *mCherry* or *PITX2c* to each cluster. **d.** UMAP plots showing the scRNA-seq clusters and heat-maps that show the distribution of relevant marker gene expression. **e**. Gene Ontology analysis of genes up and down-regulated in C0 (*PITX2c*) compared to C0 (*mCherry*). **f.** Volcano plot showing that the long non-coding RNA *PLAYRR* is downregulated in C0 (*PITX2c*) vs C0 (*mCherry*) alongside other key PC markers. **g.** Correlation of gene expression in AAV6-*PITX2c* PCM C0 and C7 with *PITX2* and *EGFP*. **h.** Correlation of gene expression in C0 and C7 with *EGFP* expression. scRNA-seq, single cell RNA sequencing; CM, cardiomyocyte; C, cluster; PE, pro-epicardium; PC, pacemaker CM; ACM, atrial CM; VCM, ventricular CM.

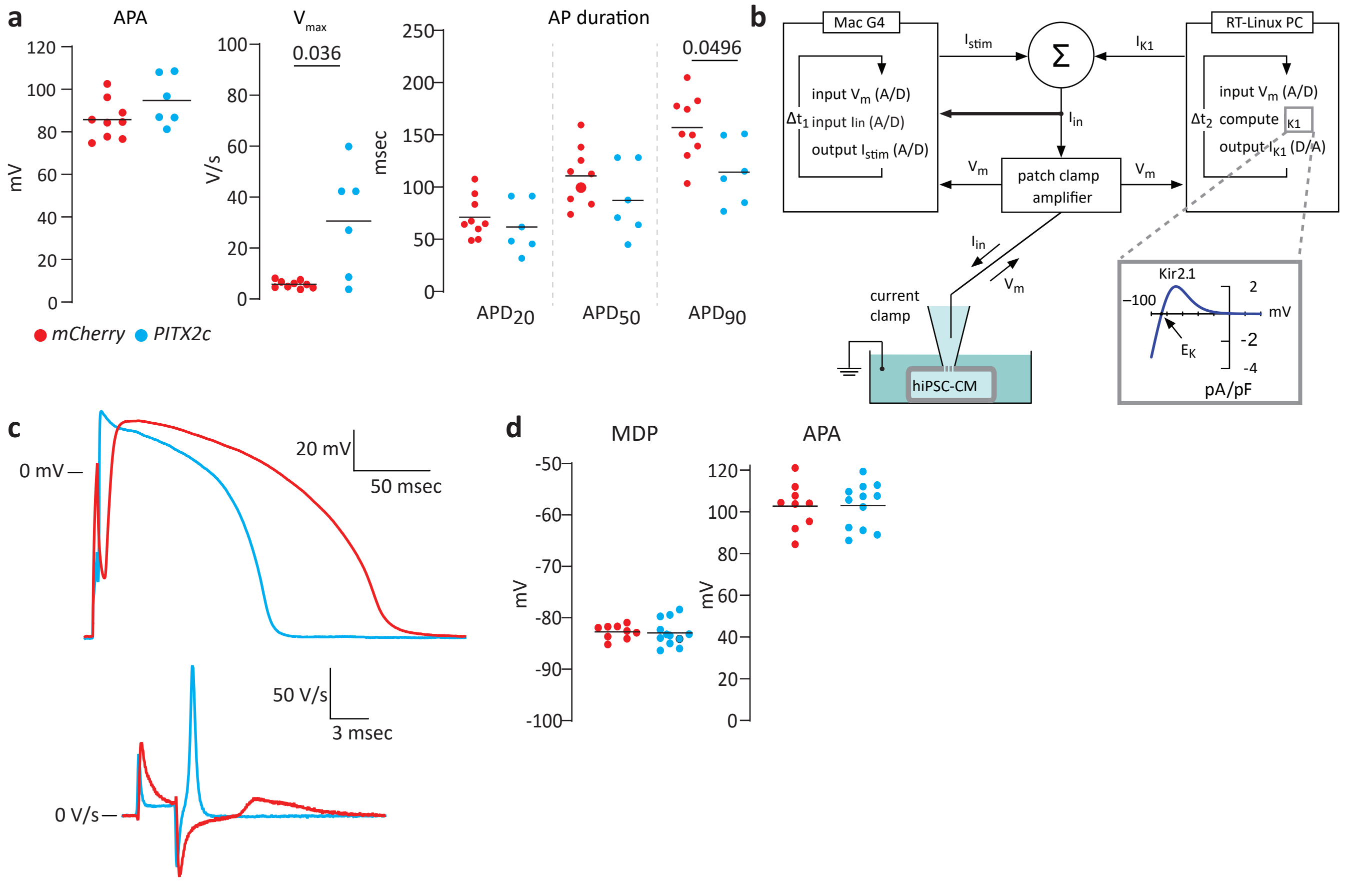

a
APA
mV
120
100
80
60
40
20
0
mCherry
PITX2c
Vmax
V/s
0.036
AP duration
msec
APD20
APD50
APD90
0.0496
b
Mac G4
input Vm (A/D)
Δt1 input Iin (A/D)
output Istim (A/D)
Istim
Σ
IK1
Iin
Vm
patch clamp amplifier
RT-Linux PC
input Vm (A/D)
Δt2 compute IK1
output IK1 (D/A)
current clamp
hiPSC-CM
Kir2.1
−100
mV
EK
2
-2
-4
pA/pF
c
0 mV
20 mV
50 msec
50 V/s
3 msec
0 V/s
d
MDP
APA
mV
-50
-60
-70
-80
-90
-100

Supplementary Figure 7. Electrophysiological changes in hiPSC-derived pacemaker-like cells upon *PITX2c* overexpression. a. Dot plot showing that the APA is comparable, the AP upstroke velocity (V_{max}) is increased (two-tailed Mann-Whitney test, p=0.036) and the APD_{90} is significantly shorter in AAV6-*PITX2c* PCs (blue) compared to AAV6-*mCherry* controls (red) (two-tailed Mann-Whitney test, p=0.0496). **b.** Diagram outlining Kir2.1 injection using dynamic clamp. **c.** Typical examples of APs (top panel) and V_{max} (bottom panel) evoked at 1 Hz under dynamic clamp conditions. **d.** MDP and APA are unchanged following Kir2.1 injection in in AAV6-*PITX2c* PCs compared to AAV6-*mCherry* controls. AP parameters were established of 9 AAV6-*mCherry* control and 12 AAV6-*PITX2c* PCs. Source data are provided in the Source Data file. APA, action potential amplitude; AP, action potential; APD_{90}, action potential duration at 90% repolarization; MDP, maximal diastolic potential.

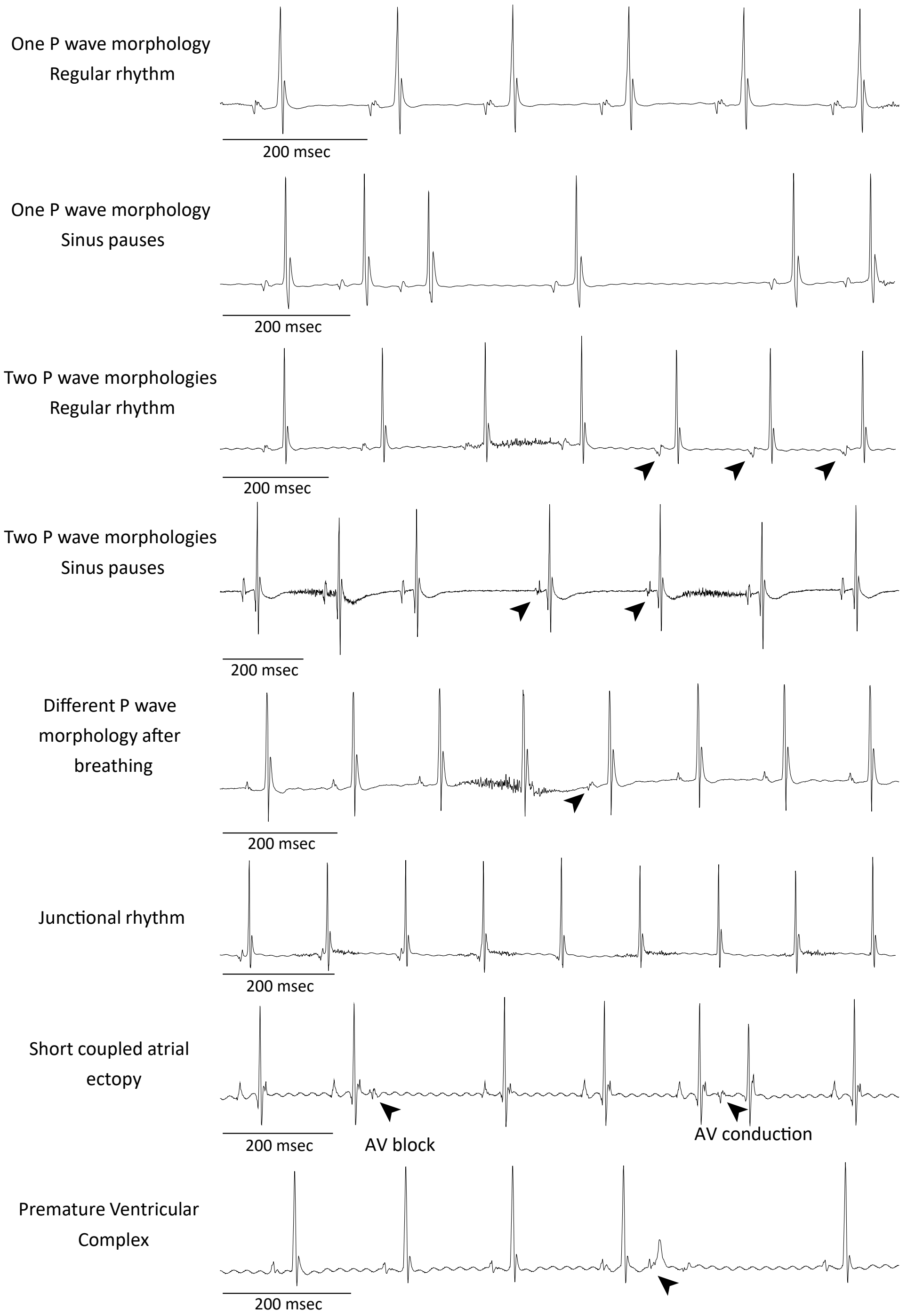

One P wave morphology
Regular rhythm
200 msec
One P wave morphology
Sinus pauses
200 msec
Two P wave morphologies
Regular rhythm
200 msec
Two P wave morphologies
Sinus pauses
200 msec
Different P wave
morphology after
breathing
200 msec
Junctional rhythm
200 msec
Short coupled atrial
ectopy
200 msec
AV block
AV conduction
Premature Ventricular
Complex
200 msec

Supplementary Figure 8. Electrocardiographic observations in *delB* mice. Several ECG phenotypes were observed in both *delB/+* and *delB/delB* adult mice. Arrows point to inverted or retrograde P waves or ventricular complexes. ECG, electrocardiogram; AV, atrioventricular.

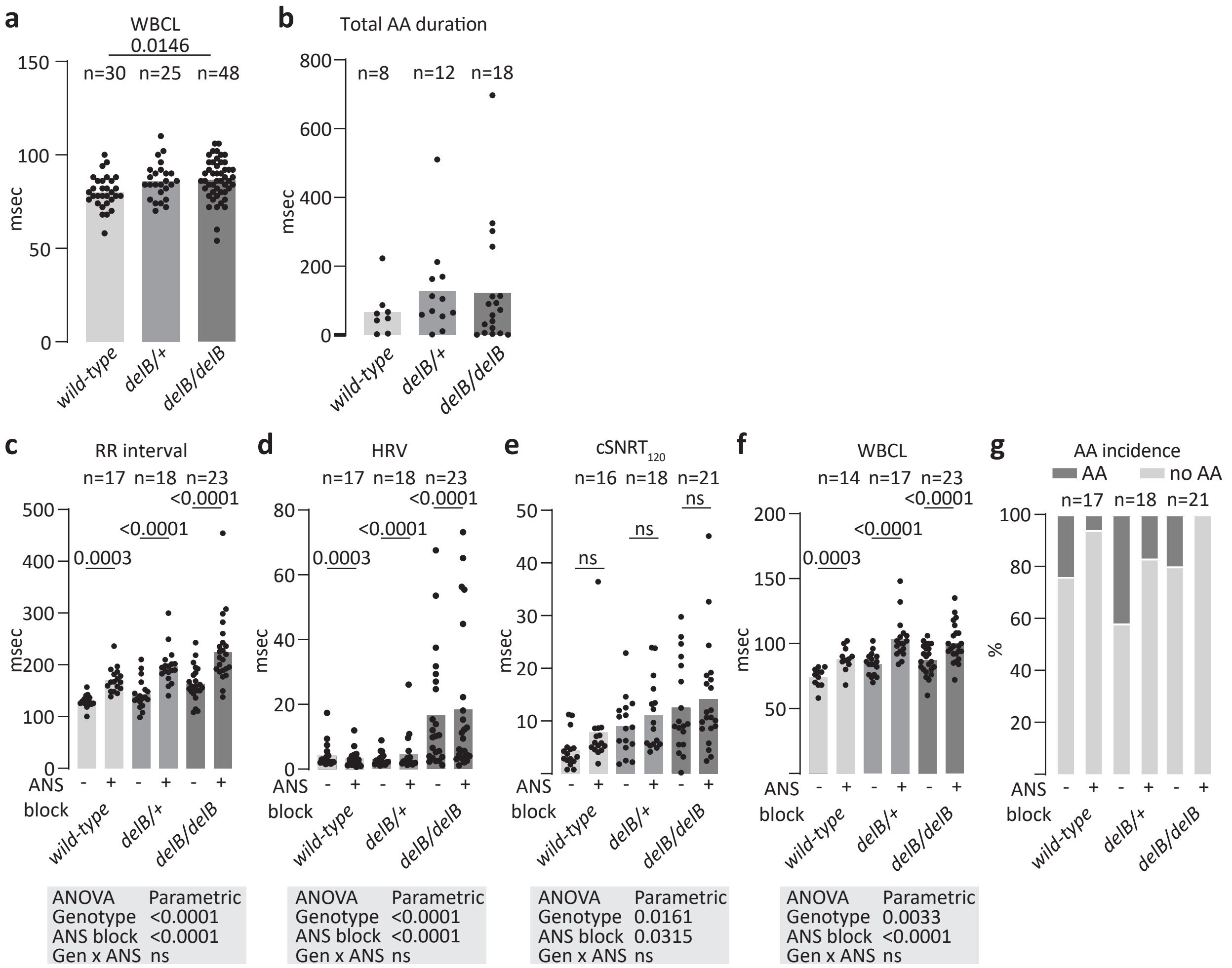
a
WBCL
0.0146
n=30 n=25 n=48
msec
wild-type
delB/+
delB/delB
b
Total AA duration
n=8 n=12 n=18
msec
c
RR interval
n=17 n=18 n=23
0.0003
<0.0001
<0.0001
ANS block
d
HRV
e
cSNRT120
n=16 n=18 n=21
ns
f
WBCL
n=14 n=17 n=23
g
AA incidence
AA
no AA
n=17 n=18 n=21
%
ANOVA Parametric
Genotype <0.0001
ANS block <0.0001
Gen x ANS ns
ANOVA Parametric
Genotype <0.0001
ANS block <0.0001
Gen x ANS ns
ANOVA Parametric
Genotype 0.0161
ANS block 0.0315
Gen x ANS ns
ANOVA Parametric
Genotype 0.0033
ANS block <0.0001
Gen x ANS ns

Supplementary Figure 9. *DelB* electrocardiographic parameters persist upon autonomic nervous system block a. WBCL was prolonged in *delB/delB* mice compared to *wild-type* mice (Kruskal-Wallis test and Dunn's multiple comparisons test). **b.** The total AA duration following incremental, rapid pacing was unchanged across all genotypes (Kruskal-Wallis test and Dunn's multiple comparisons test). **c.** RR interval was prolonged upon ANS block across all genotypes and did not change the previously determined genotype-associated phenotype (2-way ANOVA with Bonferroni's multiple comparisons test). **d.** HRV was slightly elevated upon ANS block across all genotypes and did not change the previously determined genotype-associated phenotype (2-way ANOVA with Bonferroni's multiple comparisons test). **e.** $cSNRT_{120}$ was unchanged upon ANS block across all genotypes and did not change the previously determined genotype-associated phenotype (2-way ANOVA with Bonferroni's multiple comparisons test). **f.** WBCL was prolonged upon ANS block across all genotypes. In this comparison, WBCL was significantly prolonged both before and after ANS block in *delB/delB* mice compared to *wild-type* mice (2-way ANOVA with Bonferroni's multiple comparisons test). **g.** Total AA duration was comparable between genotypes and did not change following ANS block. Source data are provided in the Source Data file. WBCL, Wenckebach cycle length; AA, atrial arrhythmia; ANS, autonomic nervous system; HRV, heart rate variation; $cSNRT_{120}$, corrected sinus node recovery time after 120 msec pacing.

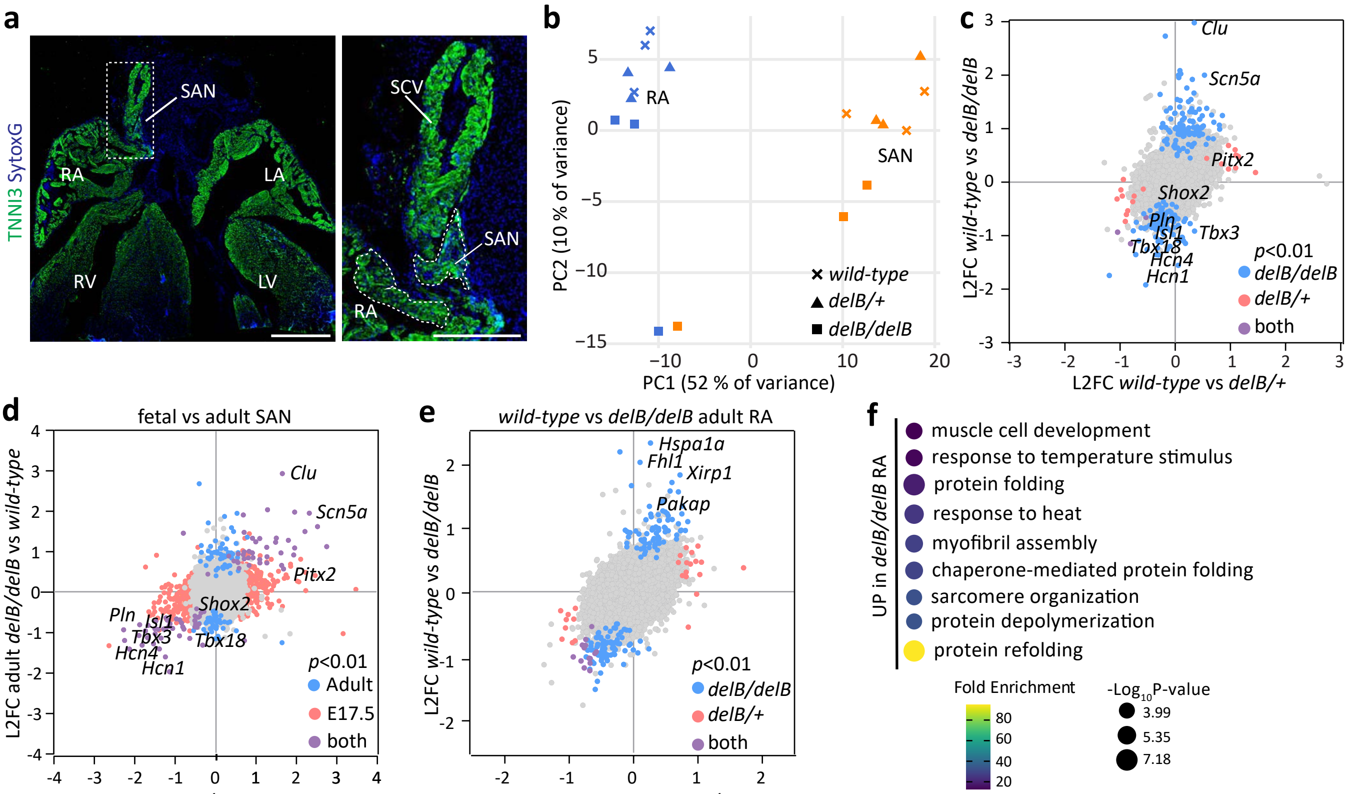

a
TNNI3 SytoxG
SAN
RA
LA
RV
LV
SCV
b
RA
SAN
PC1 (52 % of variance)
PC2 (10 % of variance)
wild-type
delB/+
delB/delB
c
Clu
Scn5a
Pitx2
Shox2
Pln
Isl1
Tbx3
Tbx18
Hcn4
Hcn1
p<0.01
delB/delB
delB/+
both
L2FC wild-type vs delB/+
L2FC wild-type vs delB/delB
d
fetal vs adult SAN
Adult
E17.5
L2FC E17.5 delB/delB vs wild-type
L2FC adult delB/delB vs wild-type
e
wild-type vs delB/delB adult RA
Hspa1a
Fhl1
Xirp1
Pakap
f
UP in delB/delB RA
muscle cell development
response to temperature stimulus
protein folding
response to heat
myofibril assembly
chaperone-mediated protein folding
sarcomere organization
protein depolymerization
protein refolding
Fold Enrichment
-Log10P-value

Supplementary Figure 10. *DelB*-induced transcriptional deregulation persists in the adult *delB* mouse. **a.** Localized gene expression changes in the adult *wild-type* (n=3), *delB/+* (n=3) and *delB/delB* (n=3) SAN and RA were defined by spatial transcriptomics using anatomical landmarks to segment regions of interest based on TNNI3 expression. **b.** Principal component analysis showing that the transcriptomic profiles of the adult SAN and RA are distinct from one another. **c.** Scatterplot showing that the adult *delB/+* SAN is much less transcriptionally affected than the *delB/delB* SAN compared to the *wild-type* SAN. Wald test corrected for multiple comparisons using the Benjamini-Hochberg method**. d.** Scatterplot showing that the fetal transcriptional deregulation in the *delB/delB* SAN largely persists into adulthood. Wald test corrected for multiple comparisons using the Benjamini-Hochberg method. **e.** Scatterplot showing that, unlike the *delB/+* RA, the adult *delB/delB* RA is transcriptionally affected. Wald test corrected for multiple comparisons using the Benjamini-Hochberg method**. f.** Gene ontology analysis showing an enrichment for cardiac-stress associated terms in the genes that are upregulated in the *delB/delB* RA compared to the *wild-type* RA (p<0.01). Exact p and p_{adj} values, L2FC, and fold enrichment values are listed in Supplementary Data 15-18. SAN, sinus node; RA, right atrium; RV, right ventricle; LA, left atrium; LV, left ventricle; PC, principal component; L2FC, Log_2Fold-change.

STAR Key Resources Table

REAGENT or RESOURCE	SOURCE	IDENTIFIER
Antibodies		
Anti-HCN4 (rabbit polyclonal) AB5808	Merck Millipore	AB_2120046
Anti-PITX2 (sheep, polyclonal) AF7388	Bio-Techne	AB_11128639
Anti-ISL1 (rabbit polyclonal) GTX102807	GeneTex	AB_11179180
Anti-TBX3(E-20) (goat, polyclonal) SC-31656	Santa Cruz Biotechnology	AB_661665
Anti-SHOX2 (mouse, monoclonal) AB55740	Abcam	AB_945451
Anti-CX40 (goat, polyclonal)	Santa Cruz Biotechnology	AB_2110468
Anti-NKX2-5(CSX1) (goat, polyclonal)	LabNed	LN2027081
Anti-cTnI (goat, polyclonal) 4T21/2	Hytest	AB_154084
Anti-TnI (mouse, monoclonal) MAB1691	Merck Millipore	AB_11212281
Anti-GFP (chicken, polyclonal) GFP-1020	AvesLabs	AB_10000240
Anti-PCM1 (rabbit, polyclonal) HPA023370	Atlas Antibodies/Bio-connect	AB_1855072
SYTOX Green Nucleic Acid Stain	Thermo Fisher Scientific	S7020
DAPI	Sigma	D9542
Bacterial and virus strains		
XL10-Gold Ultracompetent Cells	Agilent	200315
Chemicals, peptides, and recombinant proteins		
Atropine	Sigma	A0132-1G
Propranolol hydrochloride	Sigma	P0884-1G
Isoflurane	Pharmachemie B.V.	061756
XAV939	Tocris Bioscience	#3748/10
SB431542	Tocris Bioscience	#1614
PD173074	Selleck Chemicals	#1264
Retinoic acid	Sigma	R2625
CHIR99021	Axon Medchem	#1386
Activin-A	Miltenyi Biotec	#130-115-012
BMP4	R&D Systems	#314BP-010/CF
Fetal bovine Serum	Sigma	F7524
Experimental models: Cell lines		

hiPSC line (female, human)	iPSC core facility of Leiden University Medical Center	LUMC0099iCTRL04
HEK293T	ATCC	ATCC CRL-3216
Experimental models: Organisms/strains		
Mouse: *delB*, FVB/NJ	Jackson Laboratory and [50]	N/A
Oligonucleotides		
GeoMx Whole Transcriptome Atlas Mouse RNA Probes for NGS	Bruker Spatial Biology	
Pitx2c ISH probe	[2]	N/A
Gja5 ISH probe	[2]	N/A
Hcn4 ISH probe	[2]	N/A
Recombinant DNA		
AAV6-*PITX2c*-p2a-H2B-EGFP		
AAV6-*mCherry*-p2a-H2B-EGFP		
Software and algorithms		
GraphPad Prism version 10	GraphPad Software, San Diego, CA	https://www.graphpad.com/
Rstudio	RStudio, PBC, Boston, MA	http://www.rstudio.com/.
Galaxy	Galaxy	https://usegalaxy.org/
LabChart v8.1.28	ADInstruments	https://www.adinstruments.com/products/labchart
ImageJ 1.54f	[3]	https://imagej.net/software/fiji/
StarDist 2D 0.3.0	[4-6]	https://github.com/stardist/stardist-imagej

Amira 3D 2021.2	Thermo Fisher Scientific	https://www.thermofisher.com/nl/en/home/electron-microscopy/products/software-em-3d-vis/amira-software.html
PANTHER Classification System		https://pantherdb.org/tou.jsp
Other		
Matrigel	Corning	#356234
BPEL medium	[7]	
×1 TryPLE Select	Thermo Fisher Scientific	#12563011
Lenti-X™ Concentrator	Takara	# 631232
Dulbecco's Modified Eagle Medium F12	Thermo Fisher Scientific	# 11320033
GeoMx RNA Slide Prep Kit	Bruker Spatial Biology	

Supplementary references

1 Baudic, M. *et al.* TAD boundary deletion causes PITX2-related cardiac electrical and structural defects. *Nat Commun* **15**, 3380 (2024). https://doi.org/10.1038/s41467-024-47739-x

2 Mommersteeg, M. T. *et al.* Molecular pathway for the localized formation of the sinoatrial node. *Circ Res* **100**, 354–362 (2007). https://doi.org/10.1161/01.RES.0000258019.74591.b3

3 Schindelin, J. *et al.* Fiji: an open-source platform for biological-image analysis. *Nature methods* **9**, 676–682 (2012). https://doi.org/10.1038/nmeth.2019

4 Weigert, M. & Schmidt, U. in *2022 IEEE International Symposium on Biomedical Imaging Challenges (ISBIC).* 1–4.

5 Weigert, M., Schmidt, U., Haase, R., Sugawara, K. & Myers, G. in *2020 IEEE Winter Conference on Applications of Computer Vision (WACV).* 3655–3662.

6 Schmidt, U., Weigert, M., Broaddus, C. & Myers, G. in *Medical Image Computing and Computer Assisted Intervention – MICCAI 2018.* (eds Alejandro F. Frangi *et al.*) 265–273 (Springer International Publishing).

7 Ng, E. S., Davis, R., Stanley, E. G. & Elefanty, A. G. A protocol describing the use of a recombinant protein-based, animal product-free medium (APEL) for human embryonic stem cell differentiation as spin embryoid bodies. *Nat Protoc* **3**, 768–776 (2008). https://doi.org/10.1038/nprot.2008.42
