# Supplementary material for: PITX2 dosage-dependent changes in pacemaker cell state underlie sinus node dysfunction and atrial arrhythmias
Source: Nat Commun. 2025 Dec 5;16:11197. doi: 10.1038/s41467-025-66959-3 (PMC12712101; doi:10.1038/s41467-025-66959-3)
Supplement: Supplementary file 2 — Description of Additional Supplementary Files [file 41467_2025_66959_MOESM2_ESM.pdf]

**Description of Additional Supplementary Files**

Supplementary data 1: NanoString GeoMx E17.5 *wild-type* SAN vs *delB/delB* HCN4-high

Supplementary data 2: NanoString GeoMx E17.5 *wild-type* SAN vs *delB/delB* HCN4-low

Supplementary data 3: NanoString GeoMx E17.5 *delB/delB* HCN4-high vs *delB/delB* HCN4-low

Supplementary data 4: PANTHER GO term analysis E17.5 *wild-type* SAN vs *delB/delB* SAN

Supplementary data 5: PANTHER GO term analysis of NanoString GeoMx E17.5 *wild-type* vs

*delB/delB* SAN and RA cluster analysis

Supplementary data 6: NanoString GeoMx E17.5 *wild-type* SAN vs *delB/+* HCN4-high

Supplementary data 7: NanoString GeoMx E17.5 *wild-type* SAN vs *delB/+* HCN4-low

Supplementary data 8: NanoString GeoMx E17.5 *delB/+* HCN4-high vs *delB/+* HCN4-low

Supplementary data 9: NanoString GeoMx E17.5 *delB/+* HCN4-low vs *delB/delB* HCN4-low

Supplementary data 10: NanoString GeoMx E17.5 *delB/+* HCN4-high vs *delB/delB*

HCN4-high

Supplementary data 11: scRNA-seq cluster_0_*mCherry* vs cluster_0_*PITX2c*

Supplementary data 12: scRNA-seq cluster_0_*mCherry* vs cluster_7_*PITX2c*

Supplementary data 13: PANTHER GO term analysis of scRNA-seq cluster_0_*mCherry* vs

cluster_0_*PITX2c*

Supplementary data 14: NanoString GeoMx ROI information

Supplementary data 15: NanoString GeoMX adult *wild-type* SAN vs *delB/delB* SAN

Supplementary data 16: NanoString GeoMX adult *wild-type* SAN vs *delB/*+ SAN

Supplementary data 17: NanoString GeoMX adult *wild-type* RA vs *delB/delB* RA

Supplementary data 18: PANTHER GO term analysis adult *wild-type* RA vs *delB/delB* RA

Supplementary data 19: NanoString GeoMX E17.5 *wild-type* LAA vs *delB/*+ LAA

Supplementary data 20: NanoString GeoMX E17.5 *wild-type* LAA vs *delB/delB* LAA

Supplementary data 21: NanoString GeoMX E17.5 *wild-type* LAB vs *delB/*+ LAB

Supplementary data 22: NanoString GeoMX E17.5 *wild-type* LAB vs *delB/delB* LAB
