# Supplementary material for: PITX2 dosage-dependent changes in pacemaker cell state underlie sinus node dysfunction and atrial arrhythmias
Source: Nat Commun. 2025 Dec 5;16:11197. doi: 10.1038/s41467-025-66959-3 (PMC12712101; doi:10.1038/s41467-025-66959-3)
Supplement: Supplementary file 4 — Reporting Summary [file 41467_2025_66959_MOESM4_ESM.pdf]

nature portfolio

Corresponding author(s): Vincent Christoffels

Last updated by author(s): Aug 25, 2025

Reporting Summary

Nature Portfolio wishes to improve the reproducibility of the work that we publish. This form provides structure for consistency and transparency in reporting. For further information on Nature Portfolio policies, see our Editorial Policies and the Editorial Policy Checklist.

Statistics

For all statistical analyses, confirm that the following items are present in the figure legend, table legend, main text, or Methods section.

n/a	Confirmed	
☐	☒	The exact sample size (*n*) for each experimental group/condition, given as a discrete number and unit of measurement
☐	☒	A statement on whether measurements were taken from distinct samples or whether the same sample was measured repeatedly
☐	☒	The statistical test(s) used AND whether they are one- or two-sided<br>*Only common tests should be described solely by name; describe more complex techniques in the Methods section.*
☐	☒	A description of all covariates tested
☐	☒	A description of any assumptions or corrections, such as tests of normality and adjustment for multiple comparisons
☐	☒	A full description of the statistical parameters including central tendency (e.g. means) or other basic estimates (e.g. regression coefficient) AND variation (e.g. standard deviation) or associated estimates of uncertainty (e.g. confidence intervals)
☐	☒	For null hypothesis testing, the test statistic (e.g. *F*, *t*, *r*) with confidence intervals, effect sizes, degrees of freedom and *P* value noted<br>*Give P values as exact values whenever suitable.*
☒	☐	For Bayesian analysis, information on the choice of priors and Markov chain Monte Carlo settings
☒	☐	For hierarchical and complex designs, identification of the appropriate level for tests and full reporting of outcomes
☒	☐	Estimates of effect sizes (e.g. Cohen's *d*, Pearson's *r*), indicating how they were calculated

Our web collection on statistics for biologists contains articles on many of the points above.

Software and code

Policy information about availability of computer code

Data collection	No software was used for data collection.

Data analysis	Spatial transcriptomics: FASTQ files were trimmed, stitched and aligned using the Nanostring GeoMx NGS pipeline (version 2.3.3.10). The generated DCC files were processed with the R GeoMxTools package (version 3.8.0). Differential gene expression analysis was performed using limma (version 3.60.1).<br><br>scRNA-seq: Reads were mapped to Hg19 using STARsolo (version 2.7.11a). The alignment was performed using default parameters unless otherwise specified. The non-default parameters used were: Configure Chemistry Options- Cv3; Matching the Cell Barcodes to the WhiteList- Multimatching to WL is allowed for CBs with N-bases (CellRanger 3, 1MM_multi_Nbase_pseudocounts); Maximum to minimum ratio for UMI count- 10 for mCherry samples and 12.5 for PITX2c samples (based on Barcode Rank plots). Raw counts were extracted from BAM files using Seurat (Satija et al., 2015).<br><br>Image analysis: ImageJ 1.54f using StarDist 2D 0.3.0<br><br>Flow cytometry: FlowJo version 10<br><br>ECG analysis: LabChart v8.1.28<br><br>3D volumetric analysis: Amira 3D 2021.2

For manuscripts utilizing custom algorithms or software that are central to the research but not yet described in published literature, software must be made available to editors and reviewers. We strongly encourage code deposition in a community repository (e.g. GitHub). See the Nature Portfolio guidelines for submitting code & software for further information.

Data

Policy information about availability of data

All manuscripts must include a data availability statement. This statement should provide the following information, where applicable:

- Accession codes, unique identifiers, or web links for publicly available datasets
- A description of any restrictions on data availability
- For clinical datasets or third party data, please ensure that the statement adheres to our policy

scRNA-seq datasets obtained from hiPSC-PCs are available on GEO: GSE293813 [https://www.ncbi.nlm.nih.gov/geo/query/acc.cgi?acc=GSE293813]. Nanostring GeoMx-DSP datasets are available on GEO: GSE291746 [https://www.ncbi.nlm.nih.gov/geo/query/acc.cgi?acc=GSE291746] (E17.5 wild-type and delB/delB SAN and RA), GEO: GSE291749), [https://www.ncbi.nlm.nih.gov/geo/query/acc.cgi?acc=GSE291749] (E17.5 wild-type, delB/+, delB/delB SAN, RA, and LA), and GEO: GSE291752 [https://www.ncbi.nlm.nih.gov/geo/query/acc.cgi?acc=GSE291752] (adult wild-type, delB/+, and delB/delB SAN and RA). Source data are provided with this paper.

Research involving human participants, their data, or biological material

Policy information about studies with human participants or human data. See also policy information about sex, gender (identity/presentation), and sexual orientation and race, ethnicity and racism.

Reporting on sex and gender	n/a
Reporting on race, ethnicity, or other socially relevant groupings	n/a
Population characteristics	n/a
Recruitment	n/a
Ethics oversight	n/a

Note that full information on the approval of the study protocol must also be provided in the manuscript.

Field-specific reporting

Please select the one below that is the best fit for your research. If you are not sure, read the appropriate sections before making your selection.

☒ Life sciences ☐ Behavioural & social sciences ☐ Ecological, evolutionary & environmental sciences

For a reference copy of the document with all sections, see nature.com/documents/nr-reporting-summary-flat.pdf

Life sciences study design

All studies must disclose on these points even when the disclosure is negative.

Sample size	Where possible, all experiments were performed using at least 3 biological replicates. This, in combination with the consistent experimental results obtained using both human and mouse models at several different ages (and developmental stages) indicates that this minimum was sufficient.

To determine the sample size necessary to quantify differences in ECG/TEPB parameters in adult mice, we performed a power analysis based on adult wild-type and mutant mice with sinus node dysfunction (for the SNRT120 parameter):
Means:
· WT: 145 ms
· Mutant: 180 ms
SD:
· WT: 16.2
· Mutant: 34.7
Alpha: 0.05
Power: 0.8
Indicating that the required number of mice per group is 9.4.
This calculation, together with previous data (doi: 10.1161/CIRCULATIONAHA.121.054347 Figure 2A-E, doi: 10.1161/CIRCRESAHA.120.317054 Figure 5) led to us to include at least 10 mice/sex/experimental group.

Samples were allocated into experimental groups as follows,
-Mouse experiments: genotype, virus administered, sex, ANS block administration
-hiPSC-PC experiments: virus administered.

Data exclusions	No data were excluded
Replication	All attempts were succesfull
Randomization	Mice were allocated to experimental groups based on their genotype. Both male and female mice we used.
Blinding	The researcher was blinded to the genotype or administered virus during data acquisition and data analysis.

Reporting for specific materials, systems and methods

We require information from authors about some types of materials, experimental systems and methods used in many studies. Here, indicate whether each material, system or method listed is relevant to your study. If you are not sure if a list item applies to your research, read the appropriate section before selecting a response.

Materials & experimental systems

n/a	Involved in the study	
☐	☒	Antibodies
☐	☒	Eukaryotic cell lines
☒	☐	Palaeontology and archaeology
☐	☒	Animals and other organisms
☒	☐	Clinical data
☒	☐	Dual use research of concern
☐	☐	Plants

Methods

n/a	Involved in the study	
☒	☐	ChIP-seq
☐	☒	Flow cytometry
☒	☐	MRI-based neuroimaging

Antibodies

Antibodies used

Anti-HCN4 (rabbit polyclonal) AB5808 Merck Millipore AB_2120046
Anti-PITX2 (sheep, polyclonal) AF7388 Bio-Techne AB_11128639
Anti-ISL1 (rabbit polyclonal) GTX102807 GeneTex AB_11179180
Anti-TBX3(E-20) (goat, polyclonal) SC-31656 Santa Cruz Biotechnology AB_661665
Anti-SHOX2 (mouse, monoclonal) AB55740 Abcam AB_945451
Anti-CX40 (goat, polyclonal) Santa Cruz Biotechnology AB_2110468
Anti-NKX2-5(CSX1) (goat, polyclonal) LabNed LN2027081
Anti-cTnI (goat, polyclonal) 4T21/2 Hytest AB_154084
Anti-TnI (mouse, monoclonal) MAB1691 Merck Millipore AB_11212281
Anti-GFP (chicken, polyclonal) GFP-1020 AvesLabs AB_10000240
Anti-PCM1 (rabbit, polyclonal) HPA023370 Atlas Antibodies/Bio-connect AB_1855072
SYTOX Green Nucleic Acid Stain Thermo Fisher Scientific S7020
DAPI Sigma D9542

Validation

All antibodies adhere to their manufacturer's validation standards, e.g.
https://www.merckmillipore.com/deepweb/assets/sigmaaldrich/product/documents/201/990/an1785eneu-mm-mk.pdf
https://www.bio-techne.com/reagents/antibodies/antibody-validation
https://www.genetex.com/Product/Overview/primary_antibodies
https://www.abcam.com/en-us/stories/articles/how-we-validate-our-recombinant-antibodies
https://www.neobiotechnologies.com/resources/antibody-validation-methods/
https://shop.hytest.fi/product/troponin-i-cardiac-antibody

https://www.antibodiesinc.com/products/anti-green-fluorescent-protein-antibody-gfp
https://shop.bio-connect.nl/anti-pcm1-hpa023370-a90

Eukaryotic cell lines

Policy information about cell lines and Sex and Gender in Research

Cell line source(s)	hiSPC line (female, human) from the iPSC core facility of Leiden University Medical Center; LUMC0099iCTRL04<br>HEK293T from ATCC; ATCC CRL-3216
Authentication	None of the cell lines were authenticated.
Mycoplasma contamination	The cell lines tested negative for mycoplasma contamination.
Commonly misidentified lines (See ICLAC register)	These are not commonly misidentified lines.

Animals and other research organisms

Policy information about studies involving animals; ARRIVE guidelines recommended for reporting animal research, and Sex and Gender in Research

Laboratory animals	Mus musculus, FVB/NJ background (Janvier Labs)<br>Age: 12-33 weeks<br>Light regime from 07:00-19:00 (summer time); 60 lux<br>Temperature: 20-24*C<br>Relative humidity settings: 45-65%
Wild animals	No wild animals were used in this study.
Reporting on sex	Both male and female mice were used
Field-collected samples	No field-collected samples were used in this study.
Ethics oversight	The guidelines from the Directive 2010/63/EU of the European Parliament and Dutch government were followed for all animal care, housing, husbandry and experiments. All animal experimental protocols were approved by the Animal Experimental Committee of the Amsterdam University Medical Centers.

Note that full information on the approval of the study protocol must also be provided in the manuscript.

Plants

Seed stocks	*Report on the source of all seed stocks or other plant material used. If applicable, state the seed stock centre and catalogue number. If plant specimens were collected from the field, describe the collection location, date and sampling procedures.*
Novel plant genotypes	*Describe the methods by which all novel plant genotypes were produced. This includes those generated by transgenic approaches, gene editing, chemical/radiation-based mutagenesis and hybridization. For transgenic lines, describe the transformation method, the number of independent lines analyzed and the generation upon which experiments were performed. For gene-edited lines, describe the editor used, the endogenous sequence targeted for editing, the targeting guide RNA sequence (if applicable) and how the editor was applied.*
Authentication	*Describe any authentication procedures for each seed stock used or novel genotype generated. Describe any experiments used to assess the effect of a mutation and, where applicable, how potential secondary effects (e.g. second site T-DNA insertions, mosiacism, off-target gene editing) were examined.*

Flow Cytometry

Plots

Confirm that:

- [x] The axis labels state the marker and fluorochrome used (e.g. CD4-FITC).
- [x] The axis scales are clearly visible. Include numbers along axes only for bottom left plot of group (a 'group' is an analysis of identical markers).
- [x] All plots are contour plots with outliers or pseudocolor plots.
- [x] A numerical value for number of cells or percentage (with statistics) is provided.

Methodology

Sample preparation	Cultures of hiPSC-derived PCs were transduced with AAV6 particles on day 18. Virus-containing medium was removed the next day and 7 days post transduction, the cells were prepared for flow cytometry. Cells were dissociated using 1x TrypLE

	Select (Thermo Fisher Scientific #12563011) and resuspended in a buffer containing 0.5 M EDTA (Thermo Fisher Scientific #15575020) and 10% BSA (Sigma-Aldrich, #A8022).
Instrument	Acquisition was performed on FACSSymphony A1 Cell Analyzer (Beckton Dickinson
Software	Data was analyzed using FlowJo version 10.
Cell population abundance	Live cells were selected based on SSC-A and FSC-A. Single cells were then selected based on FSC-H and FSC-W.
Gating strategy	Untransduced cells were used to define the gating. Cells that were expressing higher levels of fluorescence after 488 and/or 561 excitation were considered positive cells.

☒ Tick this box to confirm that a figure exemplifying the gating strategy is provided in the Supplementary Information.
